# Supplementary material for: Evolution of sexual systems, sex chromosomes and sex-linked gene transcription in flatworms and roundworms
Source: Nat Commun. 2022 Jun 10;13:3239. doi: 10.1038/s41467-022-30578-z (PMC9187692; doi:10.1038/s41467-022-30578-z)
Supplement: Supplementary file 1 — Supplementary Information [file 41467_2022_30578_MOESM1_ESM.pdf]

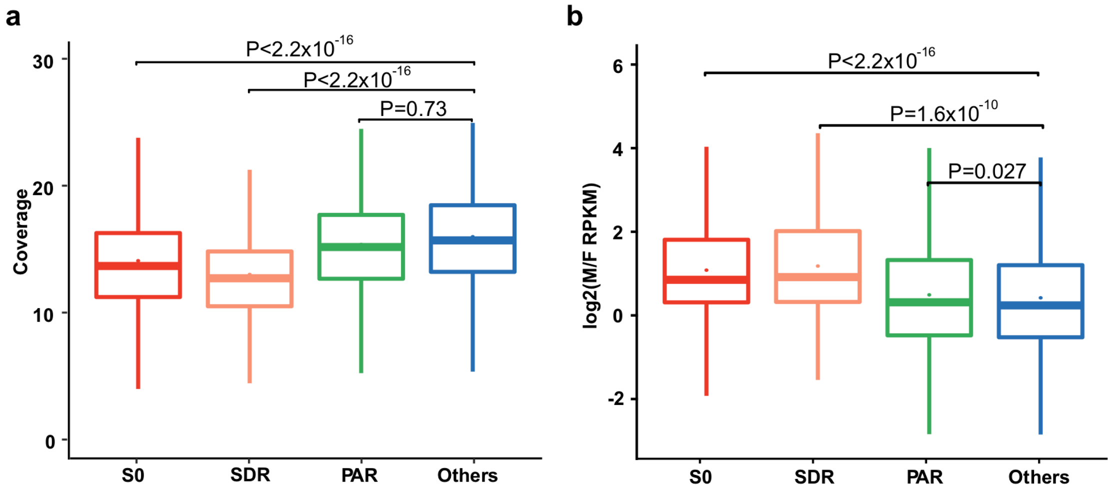

Supplementary Figure 1. *S. haematobium* (Shae) shared the ancestral evolutionary stratum (S0) with *S. mansoni*

We assigned *S. haematobium* chrZ genes to different strata based on their orthology to *S. mansoni* and *S. japonicum*. The ancestral strata shared between *S. mansoni* and *S. japonicum* was termed as S0, other strata that were specific to *S. mansoni* or *S. japonicum* also belonged to the putative SDR (sex determining region), the pseudoautosomal region (PAR) shared between *S. mansoni* and *S. japonicum* was also shown, and the rest genomic regions were shown as Others (n = 8,069). Coverage was calculated from mixed sex DNA-seq data (a), male and female RPKM values were calculated from separate male and female RNA-seq data and shown as log2(M/F RPKM) (b). Two-sided Wilcoxon rank sum tests were used for comparisons. The boxplots show the 25th percentile, median, and 75th percentile, and whiskers are set within 1.5 times the interquartile range.

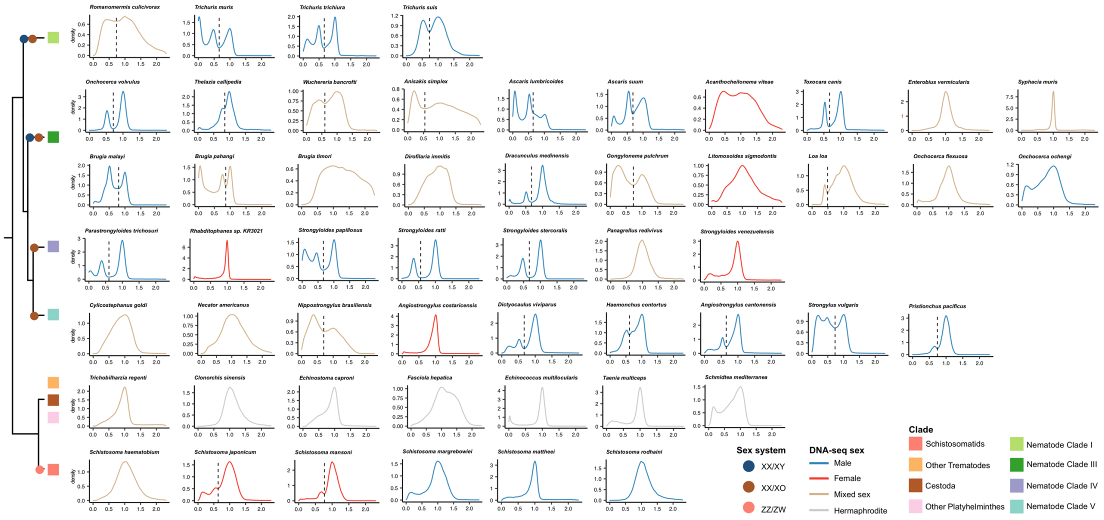

Supplementary Figure 2. Density plot of genome sequencing coverage of flatworms and roundworms.

We calculated the normalized coverage density per 10-kb window for each species shown in Figure 1a, the peak of higher coverage level that corresponds to the autosomal sequences was scaled to be centered at 1 for normalization. Different line colors represent the sex information of DNA-seq data used for genome coverage calculation. The vertical dashed line separates the candidate sex-linked sequence windows and the autosomal sequence windows. Different solid circle colors represent worm species sex chromome systems, and different solid block colors represent worm species clades.

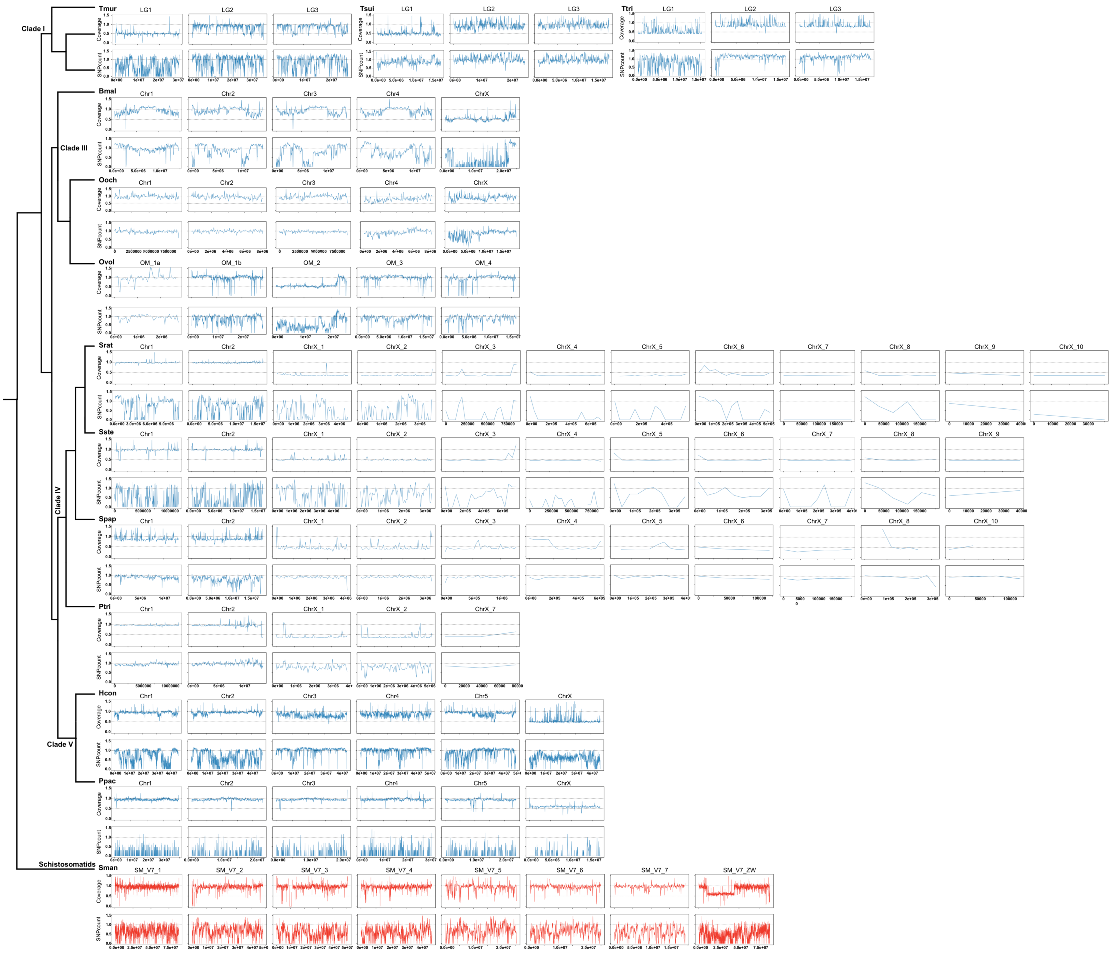

Supplementary Figure 3. Coverage and SNP density patterns of each chromosome. We calculated the normalized coverage density for each chromosome of species with chromosome-level genomes per 50 kb-long with 10-kb overlapping window. The autosomal sequences genomic coverage and SNP density were normalized to 1. Blue line represents male read coverage and red line represents female read coverage. Worm species abbreviation used in this figure: Tmur: *T. muris*, Tsui: *T. suis*, Ttri: *T. trichiura*, Bmal: *B. malayi*, Ooch: *O. ochengi*, Ovol: *O. volvulus*, Ptri: *P. trichosuri*, Spap: *S. papillosus*, Srat: *S. ratti*, Sste: *S. stercoralis*, Hcon: *H. contortus*, Ppac: *P. pacificus*, Sman: *S. mansoni*.

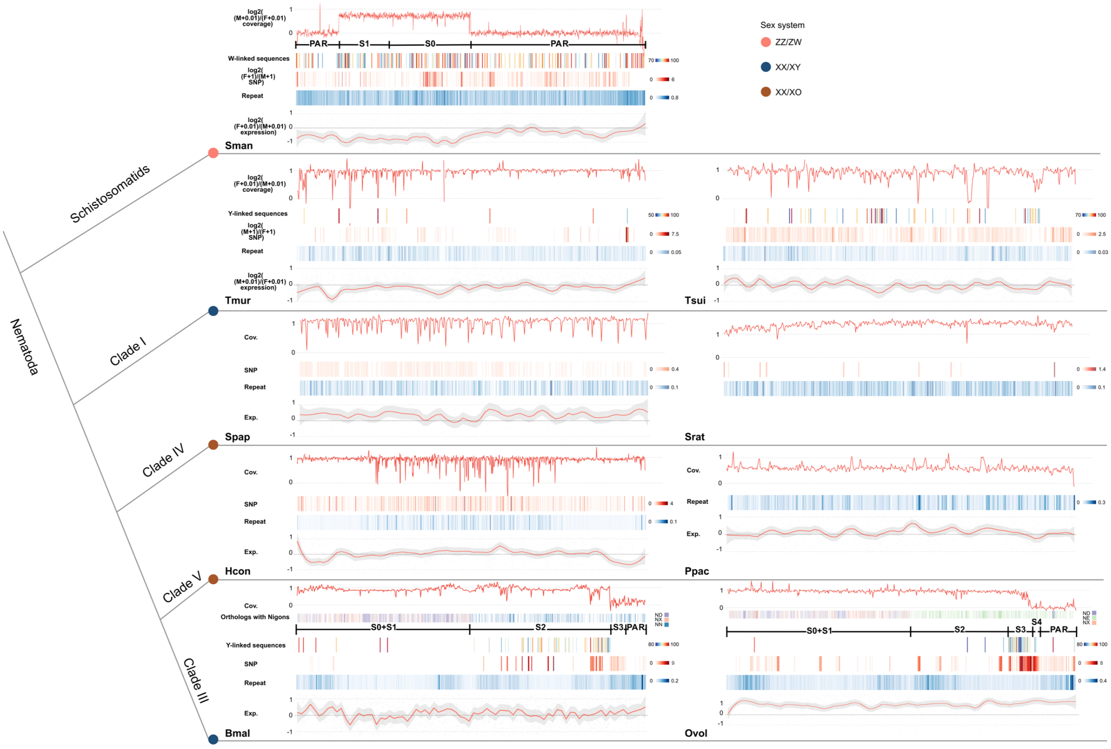

Supplementary Figure 4. Evolutionary strata of nematode sex chromosomes.
From the top to bottom we show for the log2 female vs. male coverage ratios (both coverage values add 0.01 to avoid infinity) for the XX/XO or XX/XY nematodes, and the log2 male vs. female coverage ratios for ZW system *S. mansoni*, plotted with 50-kb windows. The track “W- or Y-linked sequences” shows the assembled W- or Y-linked sequences along the chrZ or chrX, which is color-coded according to their pairwise sequence similarity levels, and shown with 50-kb windows. For *S. ratti* and *S. papillosus*, only chrX_scaffold1 is shown. The “log2((Male + 1)/(Female + 1) SNPs)” track shows the log2 ratios of male vs. female SNPs calculated per 50kb window, and windows with combined female and male SNP numbers lower than 20 were removed. The ‘Repeat’ track shows the color-scaled repeat density. And the ‘Exp.’ track shows the log2 expression ratios between sexes, calculated as male vs. female for nematodes, and female vs. male for *S. mansoni*. Sex chromosome systems are shown with different colored circles. Worm species abbreviation used in this figure: Tmur: *T. muris*, Tsui: *T. suis*, Bmal: *B. malayi*, Ovol: *O. volvulus*, Spap: *S. papillosus*, Srat: *S. ratti*, Hcon: *H. contortus*, Ppac: *P. pacificus*, Sman: *S. mansoni*. Here only shows the longest chrX scaffold of *S. ratti* and *S. papillosus*.

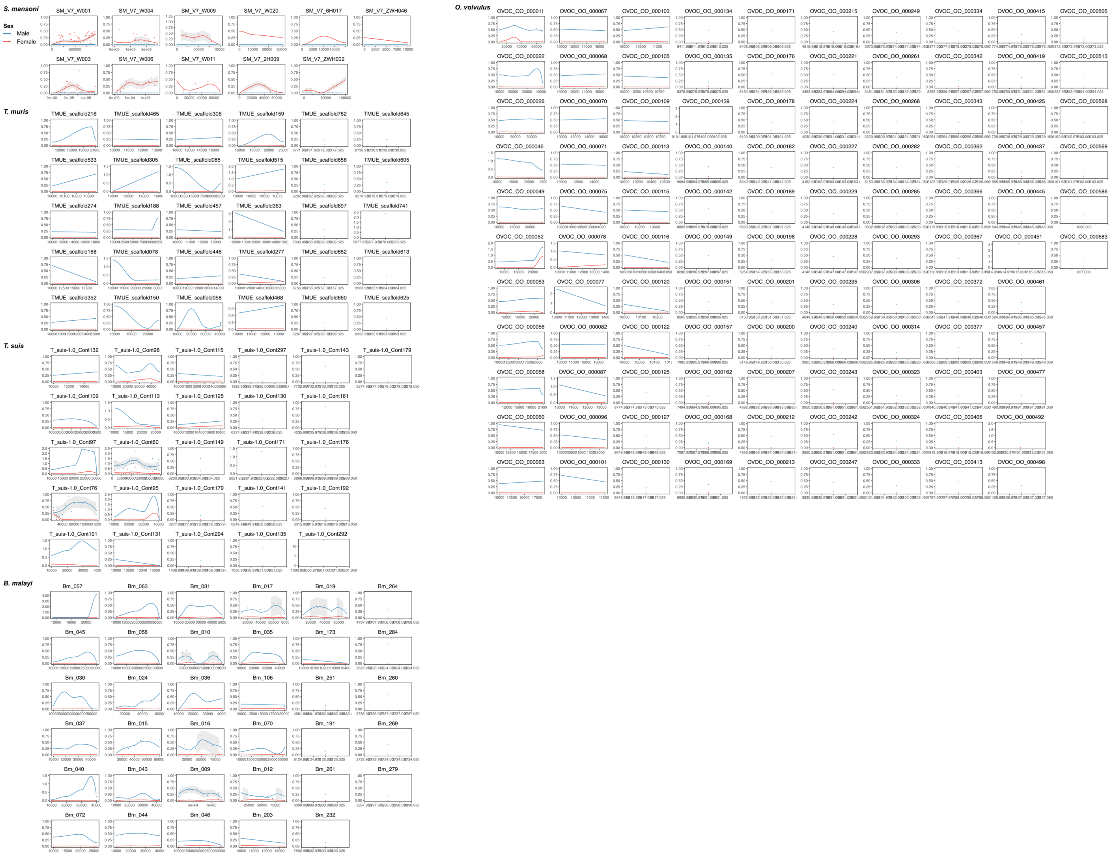

Supplementary Figure 5. Read coverage patterns of W- or Y-linked sequences.

For candidate W- or Y-linked sequences, we calculated their normalized coverage per 10-kb window. Blue line represents male read coverage, red represents female read coverage.

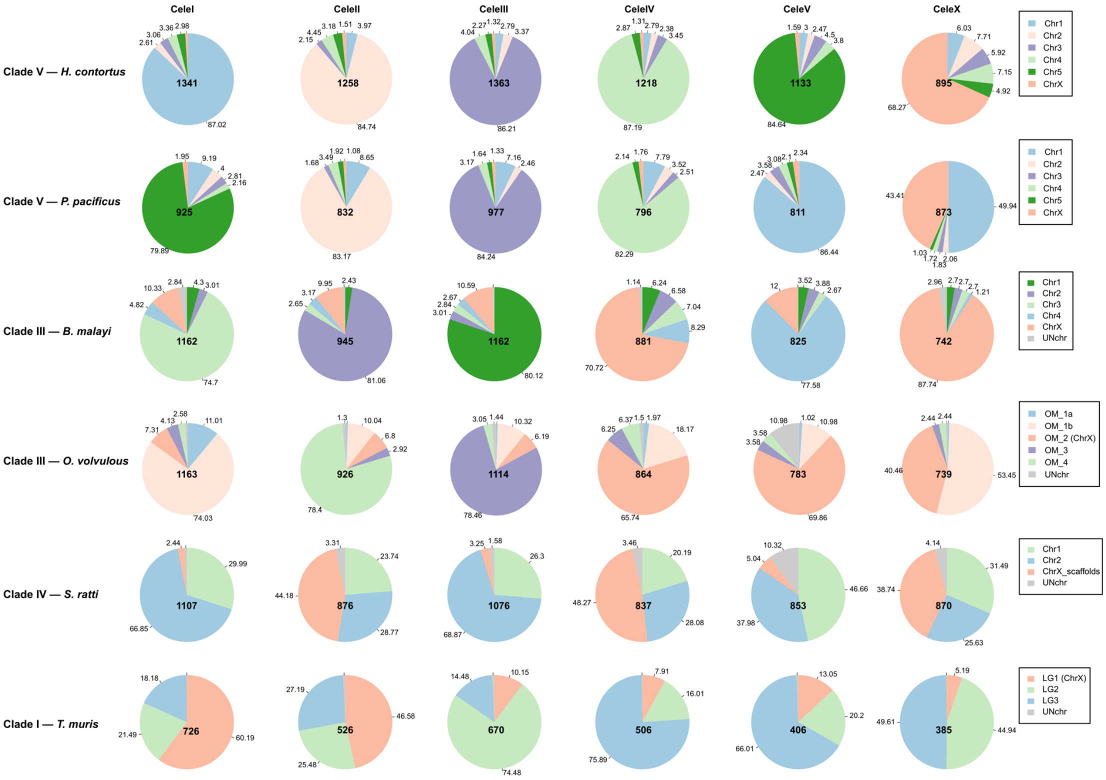

Supplementary Figure 6. The distribution of orthologous genes of *C. elegans* in other six representative nematode species with chromosome-level genomes.

The number of orthologous genes of each *C. elegans* (Cele) chromosome (I-X) in the other six species was shown as the number in the center of pie charts. Different colors represent each autosome of the other six nematode species and red represents the chrX of that species, we also showed the percentage (values >1 were shown) of orthologous genes of each chromosome outside the pie chart.

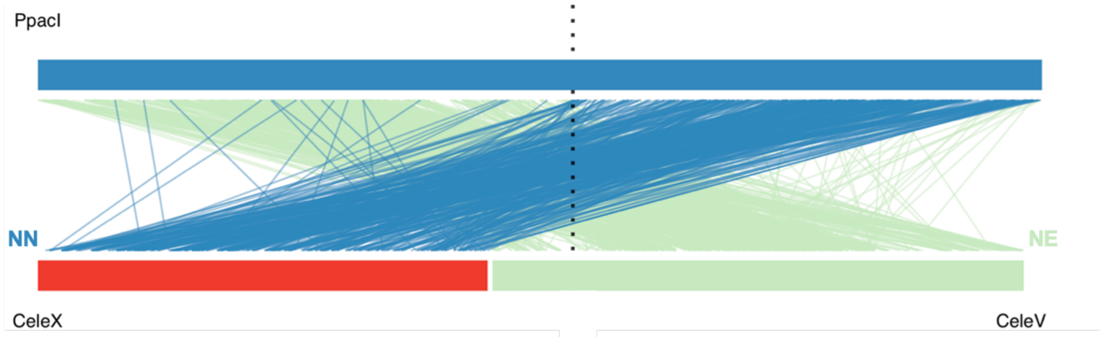

Supplementary Figure 7. Nigon elements NE and NN discrimination.
C. elegans (Cele) chrX is composed of NX and NN element[1] and its homologous genes to part of *P. pacificus* (Ppac) chrI are defined as the NN element genes. The other part of Ppac chrI is homologous to the chrV of *C. elegans* (the NE element).

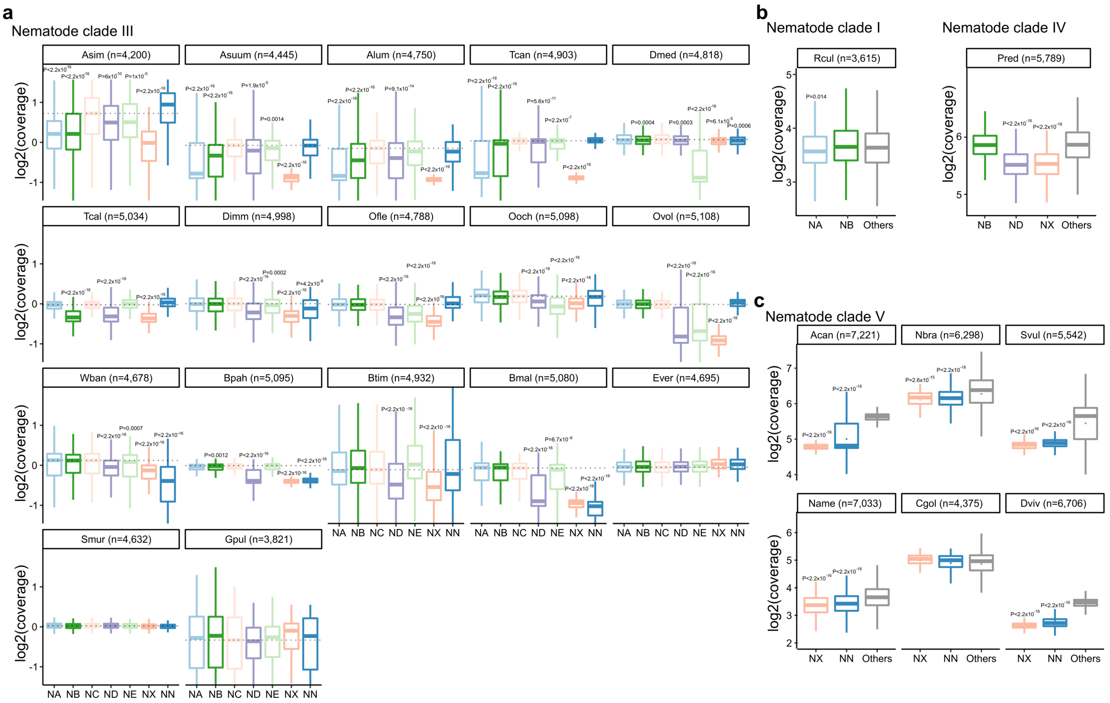

Supplementary Figure 8. Identifying the corresponding Nigon elements for the nematode sex chromosomes.

We show the male or pooled sex read coverage patterns of genes (n ranges from 3,615 to 7,221) divided by Nigon element. Nigons with a significantly lower level of read coverage (two-sided Wilcoxon ranked sum tests.) than the NC element in (a) or all the other elements in (b,c) were identified as putative sex chromosomes. The boxplots show the 25th percentile, median, and 75th percentile, and whiskers are set within 1.5 times the interquartile range. Worm species abbreviation used in this figure: Rcul: *R. culicivorax*, Pred: *P. redivivus*, Bpah: *B. pahangi*, Btim: *B. timori*, Dimm: *D. immitis*, Dmed: *D. medinensis*, Gpul: *G. pulchrum*, Ofle: *O. flexuosa*, Ooch: *O. ochengi*, Tcal: *T. callipaeda*, Wban: *W. bancrofti*, Asim: *A. simplex*, Alum: *A. lumbricoides*, Asuum: *A. suum*, Tcan: *T. canis*, Ever: *E. vermicularis*, Smur: *S. muris*, *Cgol: C. goldi*, Name: *N. americanus*, Svul: *S. vulgaris*, Acan: *A. cantonensis*, Dviv: *D. viviparus*, Nbra: *N. brasiliensis*.

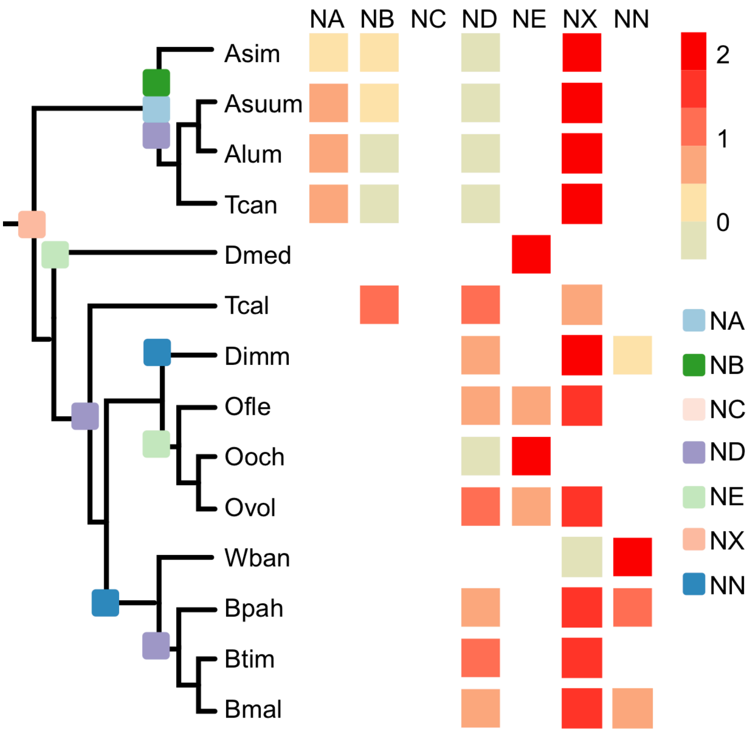

Supplementary Figure 9. Reconstructing the sex chromosome evolution in clade III species

For the clade III species, we show here the scaled percentage of sex-linked genes for each Nigon element as a heatmap. The Nigon element with at least 1% of genes as sex-linked was inferred to be part of the sex chromosomes. Worm species abbreviation used in this figure: Bmal: *B. malayi*, Bpah: *B. pahangi*, Btim: *B. timori*, Dimm: *D. immitis*, Dmed: *D. medinensis*, Ofle: *O. flexuosa*, Ooch: *O. ochengi*, Ovol: *O. volvulus*, Tcal: *T. callipaeda*, Wban: *W. bancrofti*, Asim: *A. simplex*, Alum: *A. lumbricoides*, Asuum: *A. suum*, Tcan: *T. canis*.

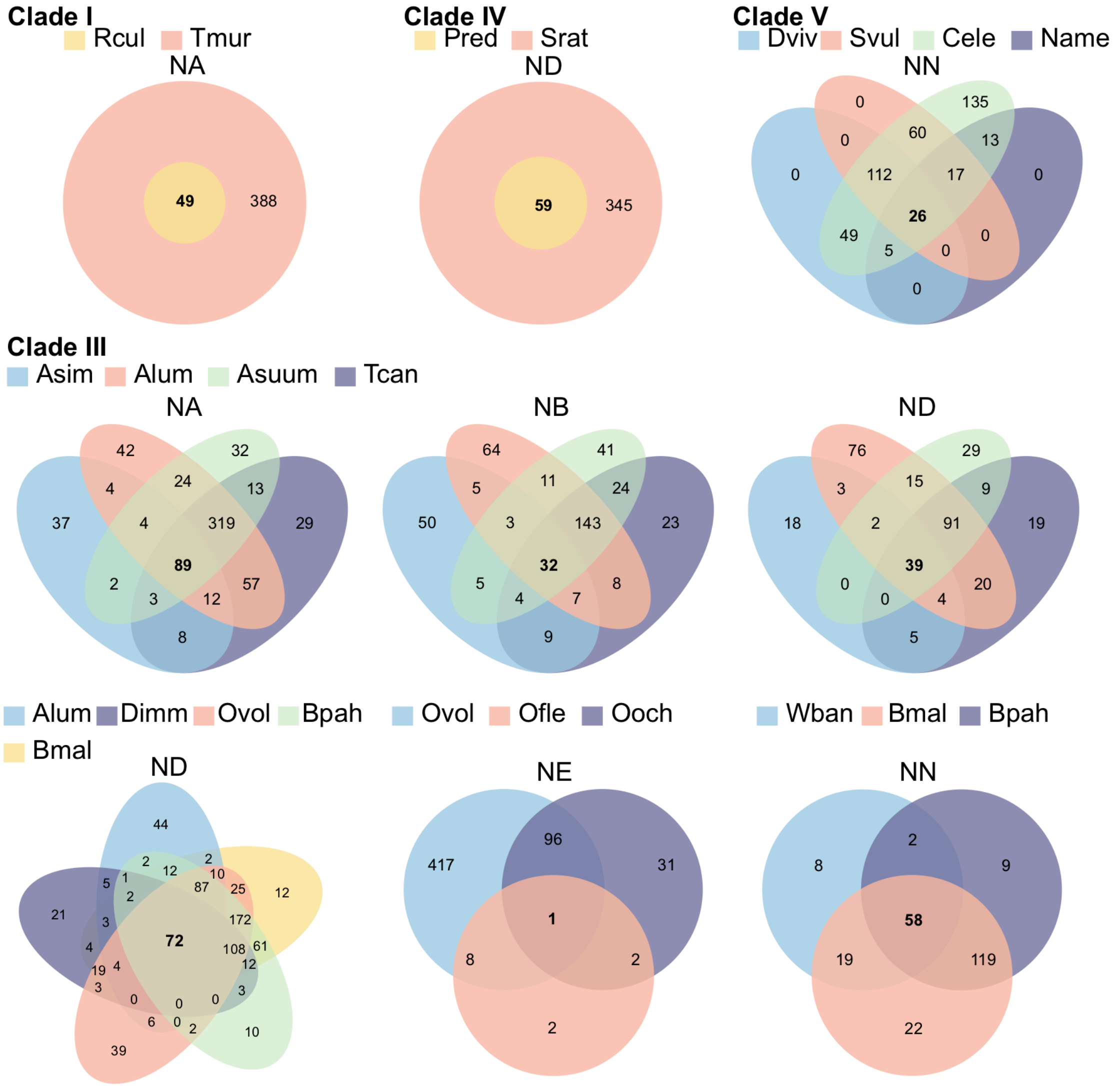

Supplementary Figure 10. Venn diagrams showing the shared sex-linked genes between different nematode species. Worm species abbreviation used in this figure: Rcul: *R. culicivorax*, Tmur: *T. muris*, Srat: *S. ratti*, Pred: *P. redivivus*, Bmal: *B. malayi*, Bpah: *B. pahangi*, Dimm: *D. immitis*, Ofle: *O. flexuosa*, Ooch: *O. ochengi*, Ovol: *O. volvulus*, Wban: *W. bancrofti*, Asim: *A. simplex*, Alum: *A. lumbricoides*, Asuum: *A. suum*, Tcan: *T. canis*, Name: *N. americanus*, Svul: *S. vulgaris*, Dviv: *D. viviparus*, Cele: *C. elegans*.

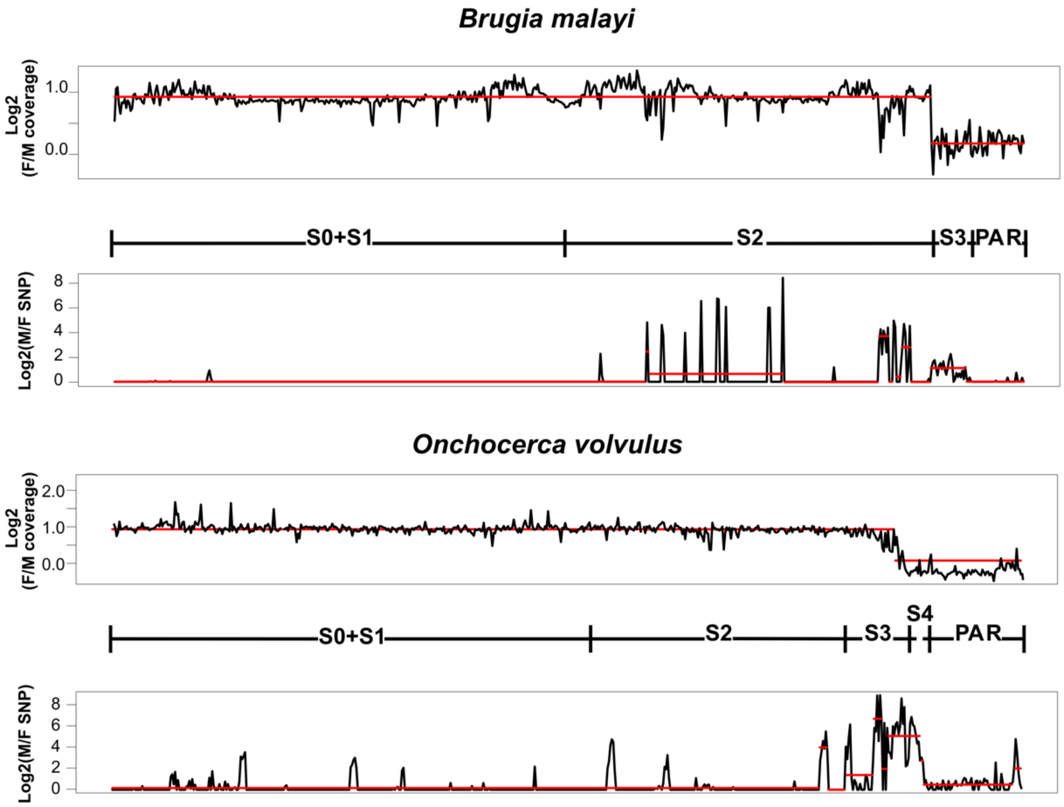

Supplementary figure 11. Change-point analysis of the coverage and SNP ratio along *B. malayi* and *O. volvulus* chrX.
Red horizontal line represents the mean value of of the coverage and SNP ratio along each segmented sequences of *B. malayi* and *O. volvulus* chrX.

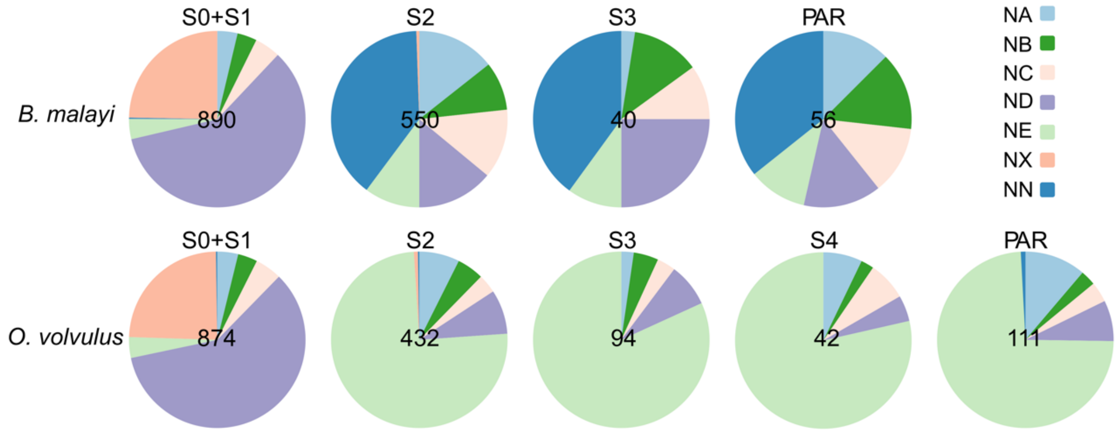

Supplementary Figure 12. The Nigon element composition of B. *malayi* and *O. volvulus* evolutionary strata and PAR.
The total homologous Nigon element gene numbers of each stratum were labeled inside the pie charts.

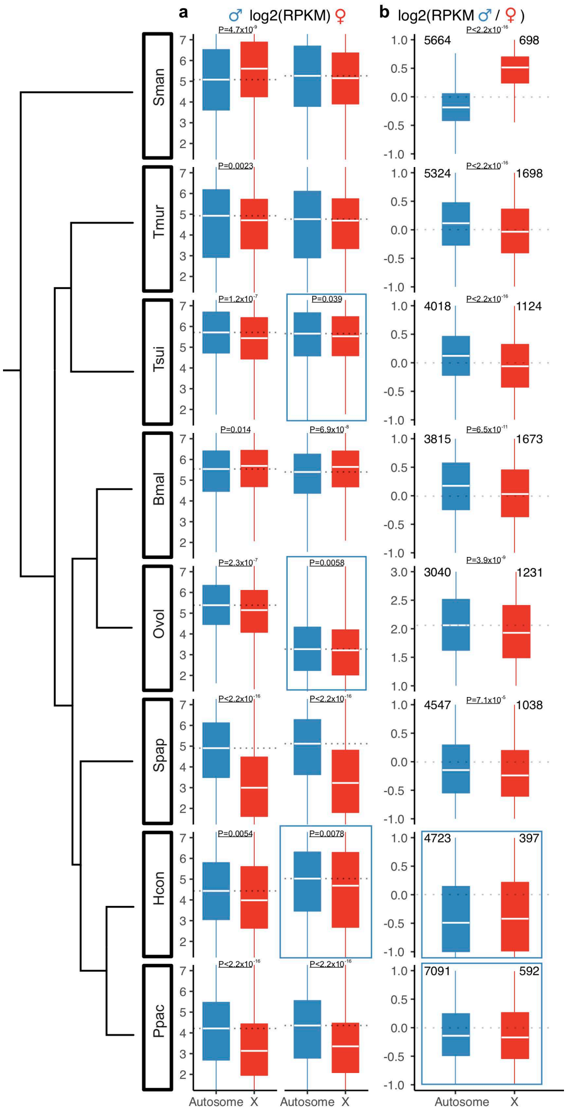

Supplementary Figure 13. Sex-linked gene expression patterns of nematodes after excluding the strongly sex-biased genes.
We defined the genes as strongly sex-biased if their expression differences between sexes were larger then 2-fold. For *O. volvuls* (Ovol), we used a cutoff of log2(Male/Female RPKM) > 3. For *H. contortus* (Hcon), we used a cutoff of log2(Female/Male RPKM) > 1.5. Only genes that show RPKM>1 in both sexes were taken into account (n ranges from 397 to 7,091). **a.** Autosomes (Autosome, blue) and chrX (X, red) of nematodes expression level in males and females. The dashed line indicates the median log2(RPKM) value of autosomes. **b.** Male and female expression ratio of autosomes (Autosome, blue) and chrX (X, red) in nematodes. Blue rectangles marked the species that exhibited a changed result of significant tests if strong sex-biased genes expression were excluded (**Figure 4**). Two-sided Wilcoxon rank sum tests were used for comparisons. The boxplots show the 25th percentile, median, and 75th percentile, and whiskers are set within 1.5 times the interquartile range. Worm species abbreviation used in this figure: Tmur: *T. muris*, Tsui: *T. suis*, Bmal: *B. malayi*, Ovol: *O. volvulus*, Spap: *S. papillosus*, Hcon: *H. contortus*, Ppac: *P. pacificus*, Sman: *S. mansoni*.

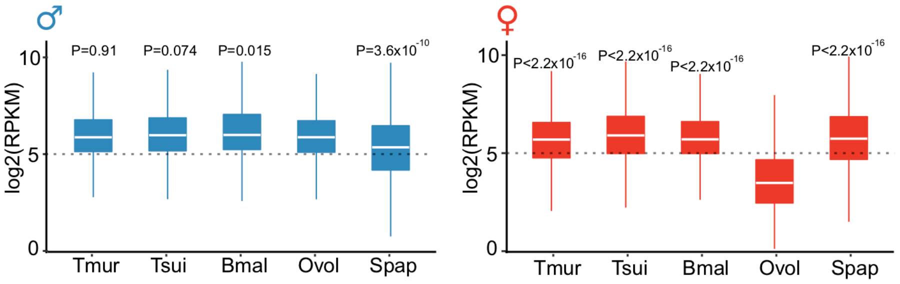

Supplementary Figure 14. Autosomal genes expression of nematode clade I, III and IV representative species in male and female. Species 1-to-1 autosomal orthologs (n= 805) log2(RPKM) comparisons used two-sided Wilcoxon rank sum tests. *O. volvulus* was used as the comparison reference. The boxplots show the 25th percentile, median, and 75th percentile, and whiskers are set within 1.5 times the interquartile range. Worm species abbreviation used in this figure: Tmur: *T. muris*, Tsui: *T. suis*, Bmal: *B. malayi*, Ovol: *O. volvulus*, Spap: *S. papillosus*.

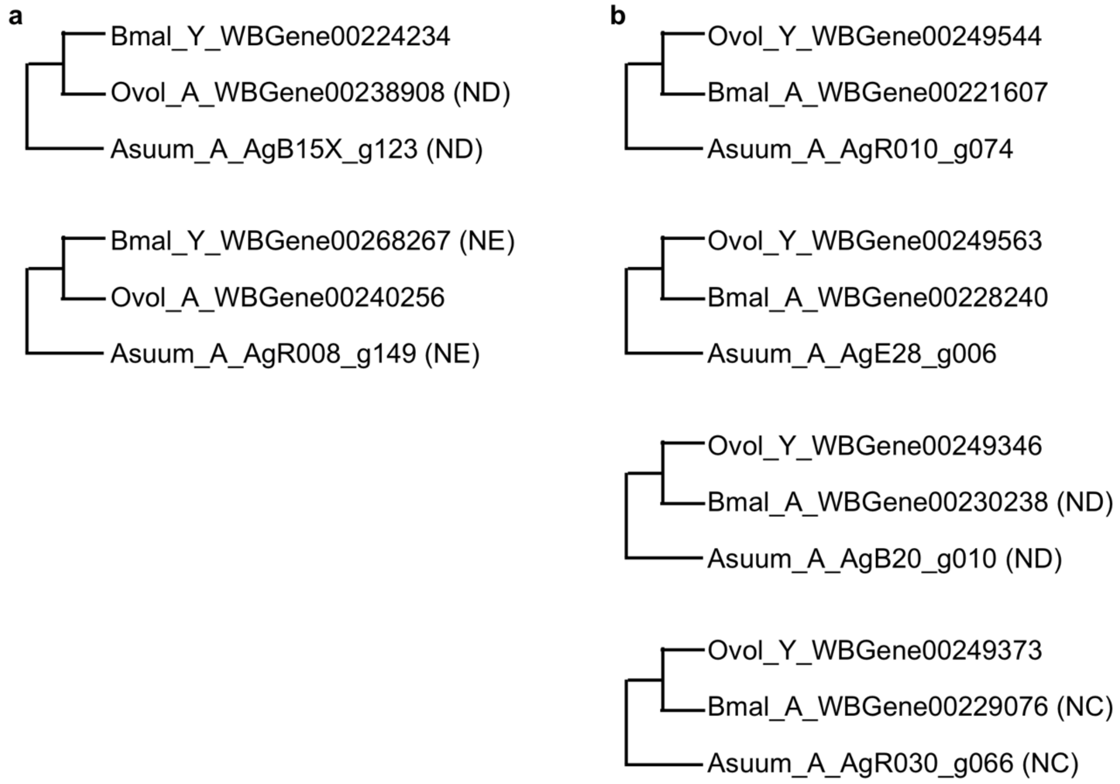

Supplementary Figure 15. Duplicated Y-linked genes derived from autosomes in *B. malayi* (a) and *O. volvulus* (b).

Phylogenetic analysis of Y-linked gene (Y) with its autosomal orthologs (A) was conducted with MEGAX (v10.1.1) using the Maximum Likelihood method. We also showed the Nigon element information for these Y-linked genes' parental genes in other related species. Worm species abbreviation used in this figure: Bmal: *B. malayi*, Ovol: *O. volvulus*, Asuum: *A. suum*.

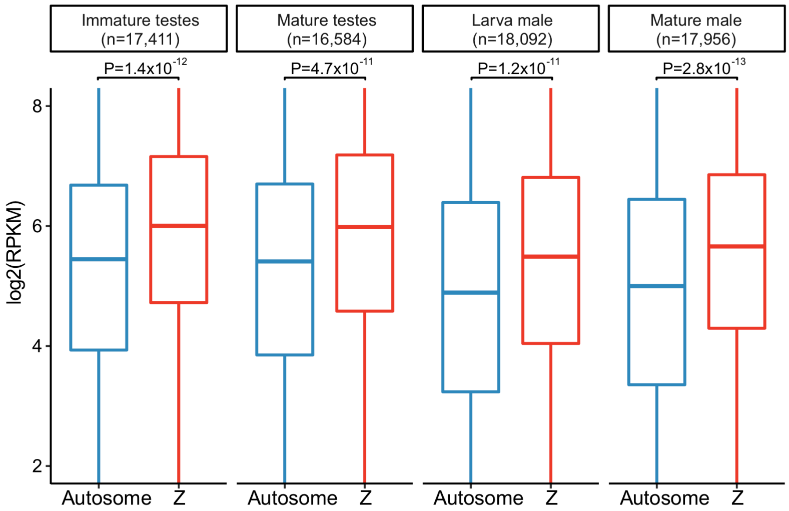

Supplementary Figure 16. Masculinization of the *S. mansoni* chrZ.
Box plots estimated *S. mansoni* genes expression on autosomes and chrZ (n ranges from 16,584 to 18,092). Only genes that show RPKM>1 were taken into account. Two-sided Wilcoxon rank sum tests were used for comparisons. The boxplots show the 25th percentile, median, and 75th percentile, and whiskers are set within 1.5 times the interquartile range.

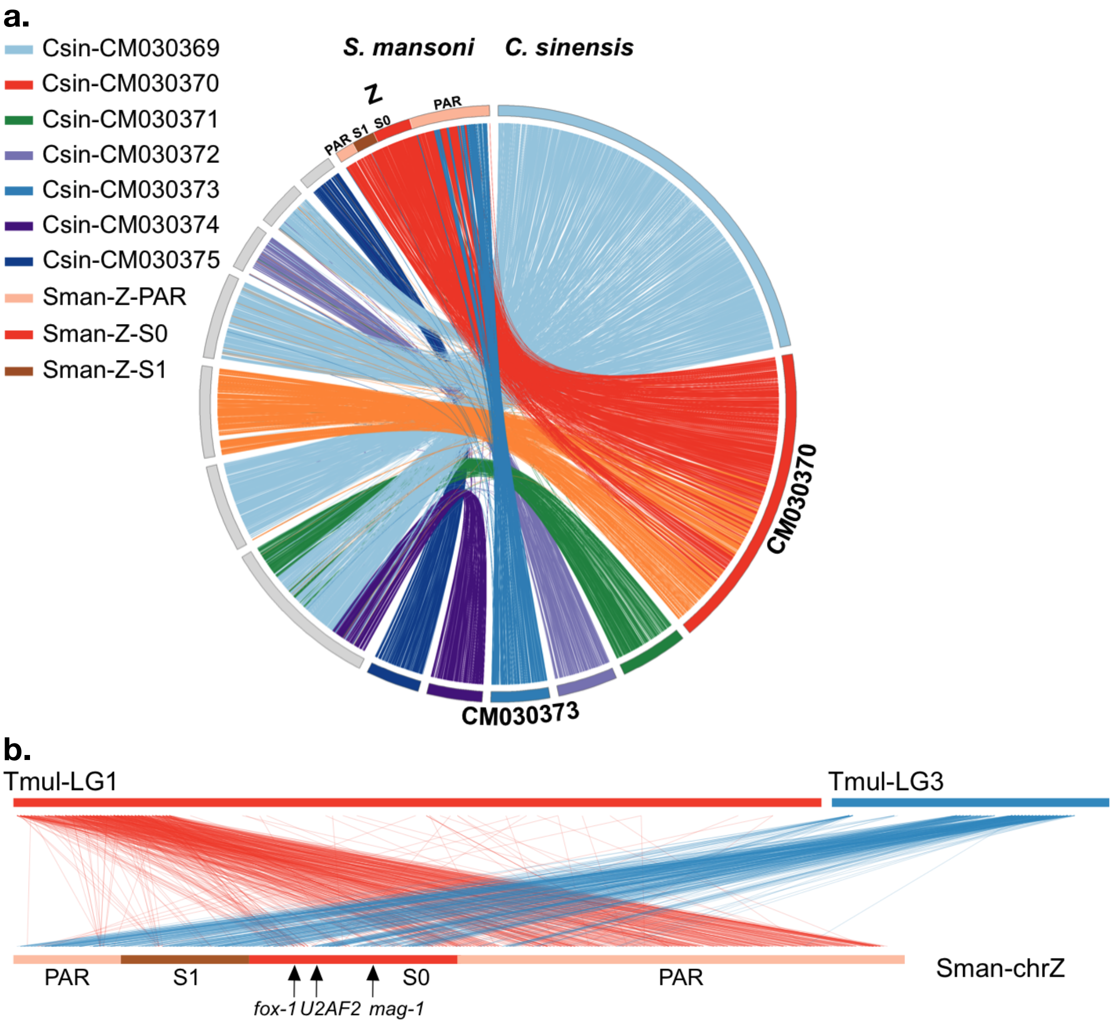

Supplementary Figure S17. Synteny relationship between *S. mansoni* chrZ and its hermaphroditic relatives *C. sinensis* (a) and *T. multiceps* (b) We also labelled the evolutionary strata and PAR of *S. mansoni* chrZ, the putative female-determining gene *U2AF2*, and the orthologs of *C. elegans* sex-determining genes *fox-1* and *mag-1*. Worm species abbreviation used in this figure: Csin: *C. sinensis*, Sman: *S. mansoni*, Tmul: *T. multiceps*.

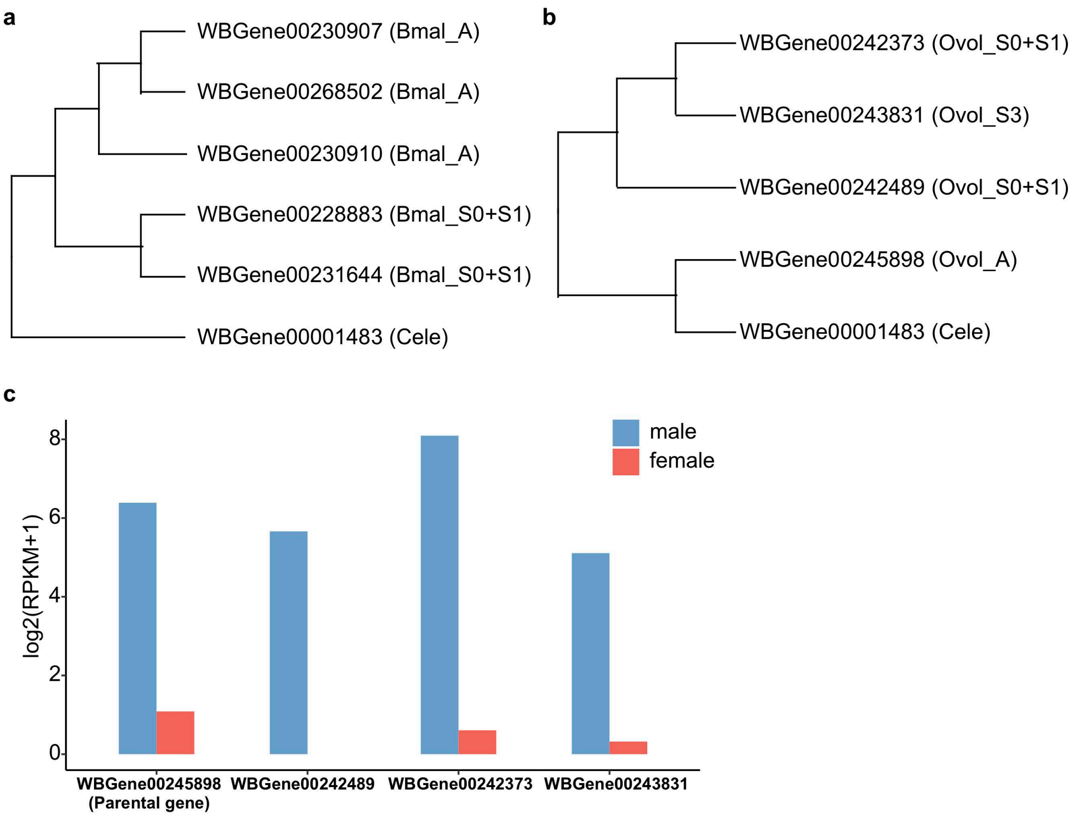

Supplementary Figure 18. Phylogenetic tree of *fog-3* homologs in *B. malayi* (a) and *O. volvulus* (b).

The other gene names in the phylogenetic tree represent *C. elegans fog-3* (WBGene00001483) homologs in *B. malayi* or *O. volvulus*. We also indicate whether each gene is autosomal (A) or in the fully X-linked evolutionary strata S0 or S2. Phylogenetic analysis of these genes was conducted with MEGAX (v10.1.1) using the Maximum Likelihood method. **c.** shows the *fog-3* orthologs expression in *O. volvulus* male (blue) and female (red) samples. Species abbreviations used in this figure: Bmal: *B. malayi*, Ovol: *O. volvulus*, Cele: *C. elegans*.

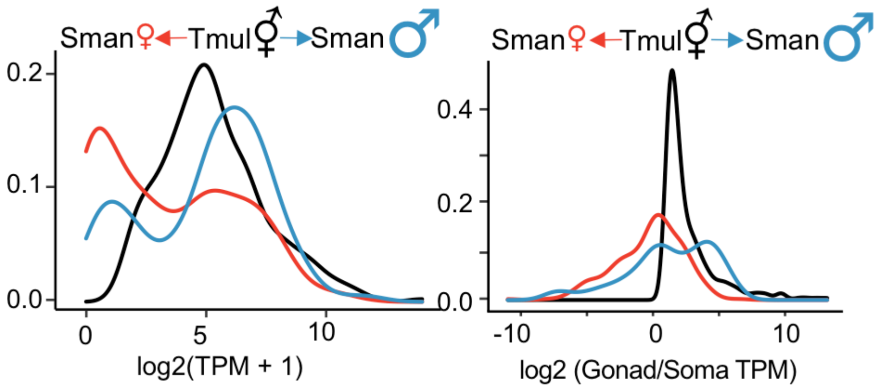

Supplementary Figure 19. Transcriptome comparison between the cestoda *Taenia multiceps* vs. *Schistosoma mansoni*.
Only *T. multiceps* (Tmul) gonad enriched genes (black colored line) were taken into account with 2-fold higher expression levels in gonads vs. soma. Their expressions were compared to either testis (blue) or ovary (red) expression of their orthologous genes in *S. mansoni* (Sman).

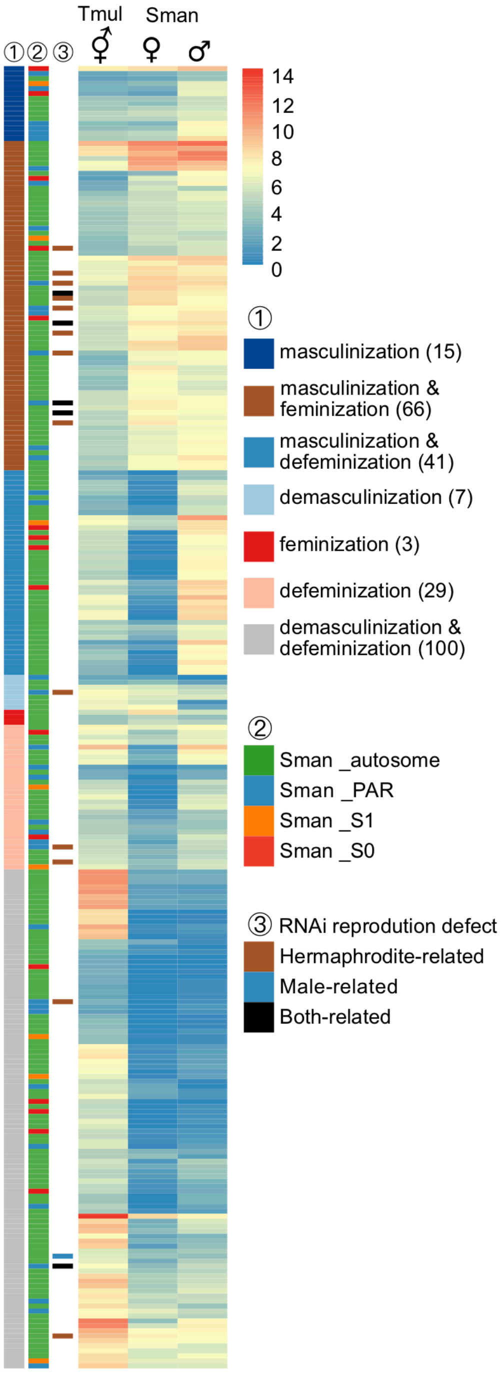

Supplementary figure 20. Comparison between the *T. multiceps* gonad enriched genes and their orthologs of *S. mansoni* for their gonad expression

Testis (♂) and ovaries (♀) of *S. mansoni* (Sman) were used for comparison of gene expression with *T. multiceps* pooled immature-mature proglottids (Tmul ☿), and we also showed their *C. elegans*' knockdown phenotype that were related to reproduction, as well as the chromosomal position of these genes in *S. mansoni*.

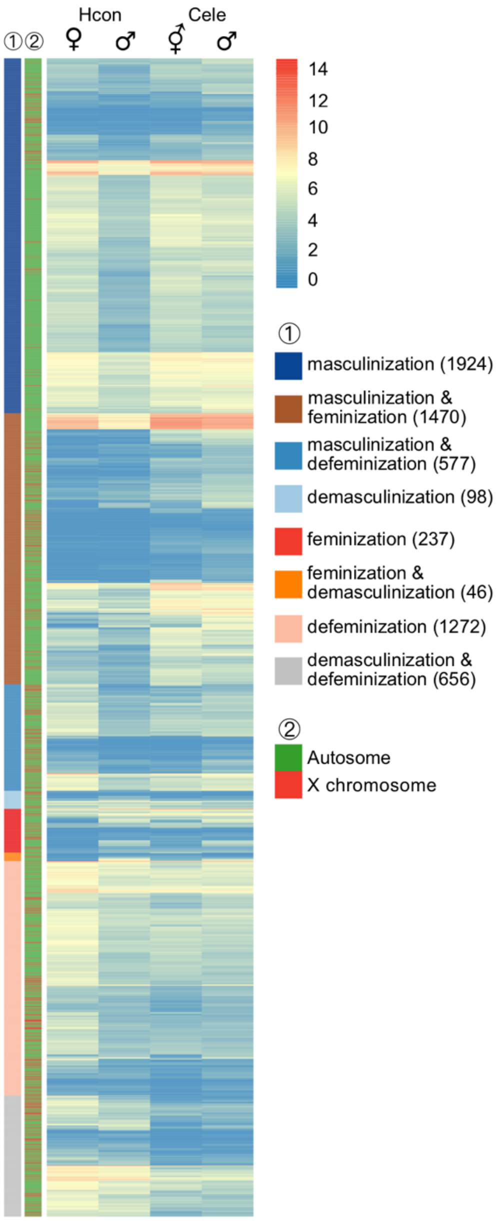

Supplementary figure 21. A 2-fold expression difference between *H. contortus* and *C. elegans*. Heatmap shows the gene expression pattern of *H. contortus* female (Hcon ♀), male (Hcon ♂), *C. elegans* hermaphrodites (Cele ⚥) and males (Cele ♂).

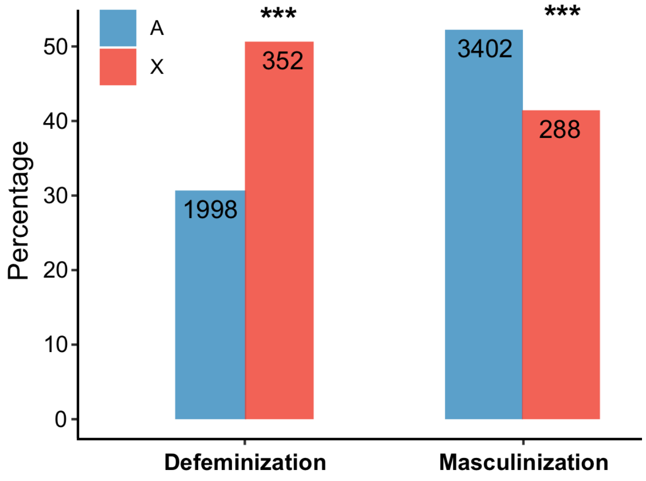

Supplementary figure 22. *C. elegans* defeminization and masculinization of chrX compared with *H. contortus*. *C. elegans* genes expression of male samples that have 2-fold higher expression (TPM) than *H. contortus* genes expression of male samples was termed as *C. elegans* masculinization genes. Gene expression of *C. elegans* hermaphrodite samples that have 2-fold lower expression (TPM) than *H. contortus* gene expression of female samples was termed as *C. elegans* defeminization genes. The defeminization and masculinization orthologs numbers are labeled, comparing autosomes (A, blue) and chrX (X, red) shows a significant accumulation of defeminization genes and a depletion of masculinization genes in *C. elegans* (n ranges from 288 to 3,402). "***" $P < 0.001$, estimated using two-sided Chi-Squared tests ($P<2.2x10^{-16}$ in both tests).

1. Tandonnet, S. *et al.* Chromosome-Wide Evolution and Sex Determination in the Three-Sexed Nematode *Auanema rhodensis*. *G3 (Bethesda)* **9**, 1211-1230 (2019).
