# Supplementary material for: Evolution of sexual systems, sex chromosomes and sex-linked gene transcription in flatworms and roundworms
Source: Nat Commun. 2022 Jun 10;13:3239. doi: 10.1038/s41467-022-30578-z (PMC9187692; doi:10.1038/s41467-022-30578-z)
Supplement: Supplementary file 3 — Description of Additional Supplementary Files [file 41467_2022_30578_MOESM3_ESM.pdf]

Description of Additional Supplementary Files

File Name: Supplementary Data 1
Description: Publicly available worm species genome assemblies employed in this project downloaded from WormBase ParaSite (https://parasite.wormbase.org/ftp.html).
The protein, CDS transcripts or canonical gene sets annotation files release version for each species we downloaded is the same with its assembly file. Published 11 chromosome-level genome assemblies were available with bold font. For *Trichuris muris*, its longest three sequences summed length is probably 82% of the whole assembly, so we termed its assembly as chromosome-level here.

File Name: Supplementary Data 2
Description: Publicly available worm species DNA/RNA-seq data employed in this project.
The species sequencing libraries information is listed.

File Name: Supplementary Data 3
Description: Improved contig- or scaffold-level genome assemblies with RaGOO in this project.
RaGOO was performed on these worm species assemblies using chromosome-level genome assemblies of their phylogenetically closely related species as references. We only retained those RaGOO output assemblies, whose genome size were larger than 60% of the input genome size.

File Name: Supplementary Data 4
Description: Worm species X- or Z-linked regions length and Y- or W-linked genes information.
Species with newly identified the elements carrying sex-linked regions in this study were with bold font. X- or Z- linked regions were identified by genomic coverage, and Y- or W-linked genes are identified by genomic coverage and Trinity v2.4.0.

File Name: Supplementary Data 5
Description: Nigon element ortholog numbers in worm species.
The species Niogn element related gene numbers are listed.

File Name: Supplementary Data 6
Description: *B. malayi* and *O. volvulus* strata information.
The numbers of gametologs in some strata are too low to estimate X-Y divergence, the KS exhibited almost no significant difference between strata, so we omitted this part.

File Name: Supplementary Data 7
Description: Autosomes or chrX gene numbers used in Figure 4a and 4b.
Genes were taken into account with RPKM-male > 1 and RPKM-female > 1.

File Name: Supplementary Data 8
Description: *B. malayi* and *O. volvulus* Y-candidates annotated employing Trinity with X-linked gametologs.
The annotated candidate Y-linked trinity contigs and their X-linked gametologs and related Nigon element are listed.

File Name: Supplementary Data 9
Description: C. elegans sex determining gene orthologs in seven representative worm species.
We assigned *C. elegans* sex determining gene as different *C. elegans* Nigon element genes based on their location, from *C. elegans* chromosome I to V were corresponding to NA to NE, *C. elegans* chrX-linked genes that have orthologs on *P. pacificus* chrX were assigned as NX genes, *C. elegans* chrX-linked genes that have orthologs on *P. pacificus* chrI were assigned as NN genes, for the rest *C. elegans* chrX-linked genes, we inffered their belonged Nigon element based on their surrounding 100-kb window NX and NN genes density, and assigned the Nigon element with higher gene density to these genes. We can assign the Nigon element *C. elegans* sex determining genes orthologs in other species for the 1-1 or 1-many *C. elegans* vs. other species ortholog pairs. A represents autosome, X represents chrX, PAR represents pseudoautosomal region, S0, S1 and S2 represent different aged strata.

File Name: Supplementary Data 10
Description: *C. sinensis* gonad enriched genes with *C. elegans* orthologs information shown RNAi reproduction defection.
Genes RNAi phenotype was obtained on website (https://wormbase.org/tools/mine/simplemine.cgi).

File Name: Supplementary Data 11
Description: *S. mansoni* feminization and feminization & masculinization *C. elegans* orthologs enriched phenotype.
S. mansoni feminization and feminization & masculinization *C. elegans* orthologs enriched phenotype was obtained on website - modPhEA (http://evol.nhri.org.tw/phenome2/) by compared these genes(n=62) against rest of genes in *C. elegans* genome(n=8294) with two-sided Fisher's exact test.

File Name: Supplementary Data 12
Description: *S. mansoni* masculinization and masculinization & defeminization *C. elegans* orthologs enriched phenotype.

S. mansoni masculinization and masculinization & defeminization *C. elegans* orthologs enriched phenotype was obtained on website - modPhEA (http://evol.nhri.org.tw/phenome2/) by compared these genes (n=31) against rest of genes in *C. elegans* genome (n=8327) with two-sided Fisher's exact test.
