# Supplementary material for: Evolution of sexual systems, sex chromosomes and sex-linked gene transcription in flatworms and roundworms
Source: Nat Commun. 2022 Jun 10;13:3239. doi: 10.1038/s41467-022-30578-z (PMC9187692; doi:10.1038/s41467-022-30578-z)
Supplement: Supplementary file 5 — Reporting Summary [file 41467_2022_30578_MOESM5_ESM.pdf]

nature portfolio

Corresponding author(s): Qi Zhou

Last updated by author(s): 2022/01/20

Reporting Summary

Nature Portfolio wishes to improve the reproducibility of the work that we publish. This form provides structure for consistency and transparency in reporting. For further information on Nature Portfolio policies, see our Editorial Policies and the Editorial Policy Checklist.

Statistics

For all statistical analyses, confirm that the following items are present in the figure legend, table legend, main text, or Methods section.

n/a	Confirmed	
☐	☒	The exact sample size (*n*) for each experimental group/condition, given as a discrete number and unit of measurement
☒	☐	A statement on whether measurements were taken from distinct samples or whether the same sample was measured repeatedly
☐	☒	The statistical test(s) used AND whether they are one- or two-sided<br>*Only common tests should be described solely by name; describe more complex techniques in the Methods section.*
☒	☐	A description of all covariates tested
☒	☐	A description of any assumptions or corrections, such as tests of normality and adjustment for multiple comparisons
☐	☒	A full description of the statistical parameters including central tendency (e.g. means) or other basic estimates (e.g. regression coefficient) AND variation (e.g. standard deviation) or associated estimates of uncertainty (e.g. confidence intervals)
☐	☒	For null hypothesis testing, the test statistic (e.g. *F*, *t*, *r*) with confidence intervals, effect sizes, degrees of freedom and *P* value noted<br>*Give P values as exact values whenever suitable.*
☒	☐	For Bayesian analysis, information on the choice of priors and Markov chain Monte Carlo settings
☒	☐	For hierarchical and complex designs, identification of the appropriate level for tests and full reporting of outcomes
☒	☐	Estimates of effect sizes (e.g. Cohen's *d*, Pearson's *r*), indicating how they were calculated

Our web collection on statistics for biologists contains articles on many of the points above.

Software and code

Policy information about availability of computer code

Data collection	1. The genomic and transcriptomic data sets used in this project were retrieved from WormBase (https://parasite.wormbase.org/index.html) and NCBI (all accession numbers are included in Supplementary Data 1 and 2).<br>2. Phylogeny tree was constructed with Evolview version 2 (http://www.evolgenius.info/evolview/).<br>3. Evolutionary timescale of worm species were estimated with TimeTree (https://www.timetree.org/).<br>4. Enriched genes phenotype was obtained on website - modPhEA (http://evol.nhri.org.tw/phenome2/).<br>5. Genes RNAi phenotype was obtained on website (https://wormbase.org/tools/mine/simplemine.cgi) and WormBase (https://parasite.wormbase.org/index.html).
Data analysis	RaGOO, SAMtools v1.11, Bowtie2 v2.2.9, BEDTools v2.27.1, Tophat v2.1.1, Trinity v2.4.0, nucmer v3.23, TransDecoder (https://github.com/TransDecoder/TransDecoder), OrthoFinder v2.2.6, BWA v0.7.17, Genome Analysis Toolkit (GATK 3.8), RepeatMasker v4.07, Changepoint (v.2.2.2), HISAT2 v2.0.4, HTSeq v0.11.0, MEGAX v10.1.1<br>All custom codes used in this work are available at: https://github.com/Flyase?tab=repositories

For manuscripts utilizing custom algorithms or software that are central to the research but not yet described in published literature, software must be made available to editors and reviewers. We strongly encourage code deposition in a community repository (e.g. GitHub). See the Nature Portfolio guidelines for submitting code & software for further information.

Data

Policy information about availability of data

All manuscripts must include a data availability statement. This statement should provide the following information, where applicable:

- Accession codes, unique identifiers, or web links for publicly available datasets
- A description of any restrictions on data availability
- For clinical datasets or third party data, please ensure that the statement adheres to our policy

The genomic and transcriptomic data sets used in this project were retrieved from WormBase ParaSite (https://parasite.wormbase.org/index.html) and NCBI (https://www.ncbi.nlm.nih.gov/). A full list of accession IDs is available in the Supplementary Data 1 and 2.

Field-specific reporting

Please select the one below that is the best fit for your research. If you are not sure, read the appropriate sections before making your selection.

☐ Life sciences ☐ Behavioural & social sciences ☒ Ecological, evolutionary & environmental sciences

For a reference copy of the document with all sections, see nature.com/documents/nr-reporting-summary-flat.pdf

Ecological, evolutionary & environmental sciences study design

All studies must disclose on these points even when the disclosure is negative.

Study description	We investigate representative species of two speciose and widely distributed phyla, Platyhelminthes (flatworms including the classes Trematoda and Cestoda) and Nematoda (Nemathelminthes, the roundworms or nematodes), to reconstruct the origin and evolution of sex chromosomes, and to describe the evolution of heteromorphism and genetic degeneration and the associated evolution of dosage compensation.
Research sample	The genomic and transcriptomic worms data sets used in this project were retrieved from WormBase ParaSite (https://parasite.wormbase.org/index.html) and NCBI (all sample species name and sexes information are included in Supplementary Data 1 and 2).
Sampling strategy	These data were retrieved from WormBase ParaSite (https://parasite.wormbase.org/index.html) and NCBI, these detailed information are introduced in other relevant published studies which are listed in the Supplementary Data 1 and 2.
Data collection	These data were retrieved from WormBase ParaSite (https://parasite.wormbase.org/index.html) and NCBI, these detailed information are introduced in other relevant published studies which are listed in the Supplementary Data 1 and 2.
Timing and spatial scale	These data were retrieved from WormBase ParaSite (https://parasite.wormbase.org/index.html) and NCBI, these detailed information are introduced in other relevant published studies which are listed in the Supplementary Data 1 and 2.
Data exclusions	No data were excluded from the analyses.
Reproducibility	Most of these samples sequencing libraries have (39 of of the 54 studied species have a rate of uniquely mapped reads higher than 90%, see Supplementary Data 2). These sequencing data were also applicable in the relevant published studies (Supplementary Data 1 and 2).
Randomization	We didn't allocate any sequencing library into groups, so randomization is not applicable in this study.
Blinding	The blinding is not not relevant in this study cause all sequenced data were processed by the same pipeline.

Did the study involve field work? ☐ Yes ☒ No

Reporting for specific materials, systems and methods

We require information from authors about some types of materials, experimental systems and methods used in many studies. Here, indicate whether each material, system or method listed is relevant to your study. If you are not sure if a list item applies to your research, read the appropriate section before selecting a response.

Materials & experimental systems

n/a	Involved in the study
☒	☐ Antibodies
☒	☐ Eukaryotic cell lines
☒	☐ Palaeontology and archaeology
☒	☐ Animals and other organisms
☒	☐ Human research participants
☒	☐ Clinical data
☒	☐ Dual use research of concern

Methods

n/a	Involved in the study
☒	☐ ChIP-seq
☒	☐ Flow cytometry
☒	☐ MRI-based neuroimaging
